# Tracing the First Days of the SARS-CoV-2 Pandemic in Greece and the Role of the First Imported Group of Travelers

**DOI:** 10.1128/spectrum.02134-22

**Published:** 2022-11-21

**Authors:** Maria Bousali, Vasiliki Pogka, Giannis Vatsellas, Theodoros Loupis, Emmanouil I. Athanasiadis, Katerina Zoi, Dimitris Thanos, Dimitrios Paraskevis, Andreas Mentis, Timokratis Karamitros

**Affiliations:** a Bioinformatics and Applied Genomics Unit, Department of Microbiology, Hellenic Pasteur Institute, Athens, Greece; b Laboratory of Medical Microbiology, Department of Microbiology, Hellenic Pasteur Institute, Athens, Greece; c Greek Genome Center, Biomedical Research Foundation of the Academy of Athens (BRFAA), Athens, Greece; d Haematology Research Laboratory, Biomedical Research Foundation of the Academy of Athens (BRFAA), Athens, Greece; e Department of Hygiene Epidemiology and Medical Statistics, School of Medicine, National and Kapodistrian University of Athens, Athens, Greece; University of Sussex

**Keywords:** SARS-CoV-2, molecular epidemiology, phylogenetics, phylodynamics, transmission clusters, infectious disease surveillance

## Abstract

The first SARS-CoV-2 case in Greece was confirmed on February 26, 2020, and since then, multiple strains have circulated the country, leading to regional and country-wide outbreaks. Our aim is to enlighten the events that took place during the first days of the SARS-CoV-2 pandemic in Greece, focusing on the role of the first imported group of travelers. We used whole-genome SARS-CoV-2 sequences obtained from the infected travelers of the group as well as Greece-derived and globally subsampled sequences and applied dedicated phylogenetics and phylodynamics tools as well as in-house-developed bioinformatics pipelines. Our analyses reveal the genetic variants circulating in Greece during the first days of the pandemic and the role of the group’s imported strains in the course of the first pandemic wave in Greece. The strain that dominated in Greece throughout the first wave, bearing the D614G mutation, was primarily imported from a certain group of travelers, while molecular and clinical data suggest that the infection of the travelers occurred in Egypt. Founder effects early in the pandemic are important for the success of certain strains, as those arriving early, several times, and to diverse locations lead to the formation of large transmission clusters that can be estimated using molecular epidemiology approaches and can be a useful surveillance tool for the prioritization of nonpharmaceutical interventions and combating present and future outbreaks.

**IMPORTANCE** The strain that dominated in Greece during the first pandemic wave was primarily imported from a group of returning travelers in February 2020, while molecular and clinical data suggest that the origin of the transmission was Egypt. The observed molecular transmission clusters reflect the transmission dynamics of this particular strain bearing the D614G mutation while highlighting the necessity of their use as a surveillance tool for the prioritization of nonpharmaceutical interventions and combating present and future outbreaks.

## INTRODUCTION

Severe acute respiratory syndrome coronavirus 2 (SARS-CoV-2), the etiologic agent of the coronavirus disease (COVID-19), spread rapidly to the global population, and as of the end of March, 2020, it was present in almost all countries worldwide (1). Various intrinsic viral and host factors have been proposed to play a crucial role in the observed differences in infectivity and spreading (2). For instance, strains bearing the D614G mutation have been noted to increase the prevalence of SARS-CoV-2 (3) and the host’s ACE2 distribution determines viral tropism (4, 5). An accurate understanding of the global spread of SARS-CoV-2 is of great importance for public health responses, prioritization of nonpharmaceutical interventions (NPIs), and prevention of future outbreaks (6). Thus, it is essential to backdate the genomic sequences of the first pandemic wave and decrypt the early events of the epidemic that triggered the regional outbreak. To date, molecular epidemiological analyses of full-length SARS-CoV-2 genomes have suggested that the origin of the transmission in Italy and Washington state was from different sources in Asia (7), while, at the same time in Spain, early introductions led to the dominance of specific strains, observed by the formation of large transmission clusters (8). The first case of COVID-19 (then 2019-nCoV) in Greece was reported on February 26, 2020, when the first imported case from Northern Italy was confirmed. Previous studies identified the genomic variants circulating in the capital city of Athens up to April 4, 2020 (9), the transmission dynamics of a COVID-19 outbreak in a Roma community in the city of Larissa (10) and the rates of onward transmission after the lift of travel restrictions early on summer 2020 in Greece (11). In this study, we aim to enlighten the first days of the SARS-CoV-2 pandemic in Greece. We analyzed the sequences of the viral genomes derived from a group of travelers that returned to Greece on February 27, 2020, through next-generation sequencing and applied molecular epidemiology approaches and in-house-developed bioinformatics pipelines in all the publicly available genome data in order to identify the contribution of the imported strains in the course of the pandemic in Greece.

## RESULTS

### Epidemiology of SARS-CoV-2 in Greece during the first pandemic wave.

On February 27, 2020, a group of 54 travelers from multiple regions of Greece returned from their trip to Mount Sinai (Egypt) and the Holy Land (Israel). Importantly, SARS-CoV-2 testing was not conducted in either the Israeli nor the Greek airport, so the travelers continued their trip back to their residence locations. The first confirmed case of the group was a 66-year-old male (Table 1; Seq.1_GROUP_GR), who presented severe symptoms of pneumonia during the weekend of February 27 to 28, 2020. On March 4, 2020, it was confirmed that he had contracted SARS-CoV-2. Up until March 7, 2020, 23/54 (42.6%) travelers had been diagnosed with a SARS-CoV-2 infection (Table 1; Seq.1_GROUP_GR – Seq.23_GROUP_GR).

**TABLE 1:** Demographic characteristics of the data set and taxonomic features of the isolated SARS-CoV-2 sequences, using the Pango lineage (26) and the Nextstrain clade (25) nomenclature

Case ID	Collection date	Patient age	Gender	Pango lineage	Nextstrain clade	GISAID accession
Imported group from Egypt and Israel to Greece						
Seq.1_GROUP_GR	2020-03-04	66	Male	B.1	20A	EPI_ISL_15424734
Seq.2_GROUP_GR	2020-03-04	44	Female	B.1	20A	EPI_ISL_15424735
Seq.3_GROUP_GR	2020-03-04	66	Female	B.1	20A	EPI_ISL_15424736
Seq.4_GROUP_GR	2020-03-05	66	Female	B.1	20A	EPI_ISL_15424737
Seq.5_GROUP_GR	2020-03-05	73	Female	B.1	20A	EPI_ISL_15424738
Seq.6_GROUP_GR	2020-03-05	57	Female	B.1	20A	EPI_ISL_15424739
Seq.7_GROUP_GR	2020-03-05	30	Female	B.1	20A	EPI_ISL_15424740
Seq.8_GROUP_GR	2020-03-05	73	Female	B.1	20A	EPI_ISL_15424741
Seq.9_GROUP_GR	2020-03-05	50	Male	B.1	20A	EPI_ISL_15424742
Seq.10_GROUP_GR	2020-03-05	NA^*a*^	Female	B.1	20A	EPI_ISL_15424743
Seq.11_GROUP_GR	2020-03-05	56	Female	B.1	20A	EPI_ISL_15424744
Seq.12_GROUP_GR	2020-03-05	52	Female	B.1	20A	EPI_ISL_15424745
Seq.13_GROUP_GR	2020-03-05	59	Male	B.1	20A	EPI_ISL_15424746
Seq.14_GROUP_GR	2020-03-05	66	Female	B.1	20A	EPI_ISL_15424747
Seq.15_GROUP_GR	2020-03-05	59	Female	B.1	20A	EPI_ISL_15424748
Seq.16_GROUP_GR	2020-03-05	68	Female	B.1	20A	EPI_ISL_15424749
Seq.17_GROUP_GR	2020-03-05	79	Male	B.1	20A	EPI_ISL_15424750
Seq.18_GROUP_GR	2020-03-05	48	Female	B.1	20A	EPI_ISL_15424751
Seq.19_GROUP_GR	2020-03-05	51	Female	B.1	20A	EPI_ISL_15424752
Seq.20_GROUP_GR	2020-03-05	80	Female	B.1	20A	EPI_ISL_15424753
Seq.21_GROUP_GR	2020-03-05	58	Male	B.1	20A	EPI_ISL_15424754
Seq.22_GROUP_GR	2020-03-05	49	Male	B.1	20A	EPI_ISL_15424755
Seq.23_GROUP_GR	2020-03-07	67	Female	B.1	20A	EPI_ISL_15424761
Subsampling from Greece						
Seq.24_GR	2020-03-05	43	Male	B.1	20A	EPI_ISL_15424756
Seq.25_GR	2020-03-05	63	Female	B.1	20A	EPI_ISL_15424757
Seq.26_GR	2020-03-05	66	Female	B.1	20A	EPI_ISL_15424758
Seq.27_GR	2020-03-05	75	Female	B.1	20A	EPI_ISL_15424759
Seq.28_GR	2020-03-05	NA	NA	B.1	20A	EPI_ISL_437894
Seq.29_GR	2020-03-06	48	Male	B.1	20A	EPI_ISL_15424760
Seq.30_GR	2020-03-07	63	Female	B.1	20A	EPI_ISL_15424762
Seq.31_GR	2020-03-07	70	Male	B.1	20A	EPI_ISL_15424763
Seq.32_GR	2020-03-08	NA	NA	B.1	20A	EPI_ISL_437896
Seq.33_GR	2020-03-08	93	Female	B.1	20A	EPI_ISL_15424764
Seq.34_GR	2020-03-09	NA	NA	B.1	20A	EPI_ISL_434455
Seq.35_GR	2020-03-10	NA	NA	B.1	20A	EPI_ISL_437905
Seq.36_GR	2020-03-10	NA	NA	B.1	20A	EPI_ISL_437900
Seq.37_GR	2020-03-10	NA	NA	B.40	19A	EPI_ISL_437906
Seq.38_GR	2020-03-12	NA	NA	B.1.1	20B	EPI_ISL_437876
Seq.39_GR	2020-03-12	NA	NA	B.1.1	20B	EPI_ISL_434467
Seq.40_GR	2020-03-12	NA	NA	B.40	19A	EPI_ISL_437888
Seq.41_GR	2020-03-13	67	Male	B.1	20A	EPI_ISL_15424765
Seq.42_GR	2020-03-14	NA	NA	B.1.1	20B	EPI_ISL_437878
Seq.43_GR	2020-03-15	54	Female	B.1	20A	EPI_ISL_15424766
Seq.44_GR	2020-03-16	NA	NA	B.40	19A	EPI_ISL_437884
Seq.45_GR	2020-03-17	40	Male	B.40	19A	EPI_ISL_15424767
Seq.46_GR	2020-03-18	NA	NA	B.1.1	20B	EPI_ISL_427043
Seq.47_GR	2020-03-18	NA	NA	B.1	20A	EPI_ISL_437890
Seq.48_GR	2020-03-19	NA	NA	B.40	19A	EPI_ISL_434481
Seq.49_GR	2020-03-19	NA	NA	B.40	19A	EPI_ISL_437892
Seq.50_GR	2020-03-20	NA	NA	B.1.1	20B	EPI_ISL_434469
Seq.51_GR	2020-03-23	NA	NA	B.40	19A	EPI_ISL_434462
Seq.52_GR	2020-03-26	NA	NA	B.1.1	20B	EPI_ISL_434468
Seq.53_GR	2020-03-29	NA	NA	B.40	19A	EPI_ISL_434459
Seq.54_GR	2020-04-01	NA	NA	B.40	19A	EPI_ISL_437910
Seq.55_GR	2020-04-04	90	Female	B.1.1	20B	EPI_ISL_487379
Seq.56_GR	2020-04-04	NA	NA	B.40	19A	EPI_ISL_434466
Seq.57_GR	2020-04-15	NA	NA	B.1	20C	EPI_ISL_2232059
Seq.58_GR	2020-04-20	NA	NA	B.1	20C	EPI_ISL_2232060
Seq.59_GR	2020-04-23	NA	NA	B.1	20C	EPI_ISL_2232061
Seq.60_GR	2020-04-27	NA	NA	B.1	20C	EPI_ISL_2232063
Seq.61_GR	2020-05-08	NA	NA	B.1	20C	EPI_ISL_2232064
Seq.62_GR	2020-05-09	NA	NA	B.1	20C	EPI_ISL_2232066
Seq.63_GR	2020-05-10	NA	NA	B.1	20C	EPI_ISL_2232067
Seq.64_GR	2020-05-11	53	Male	B.1.1	20B	EPI_ISL_15424768
Seq.65_GR	2020-05-11	53	Male	B.1.1	20B	EPI_ISL_501235
Seq.66_GR	2020-05-11	NA	NA	B.1	20C	EPI_ISL_2232070
Seq.67_GR	2020-05-11	NA	NA	B.1	20C	EPI_ISL_2232068
Seq.68_GR	2020-05-13	NA	Female	B.1.1	20B	EPI_ISL_501236

*^a^*NA, not available.

**FIG 1:**
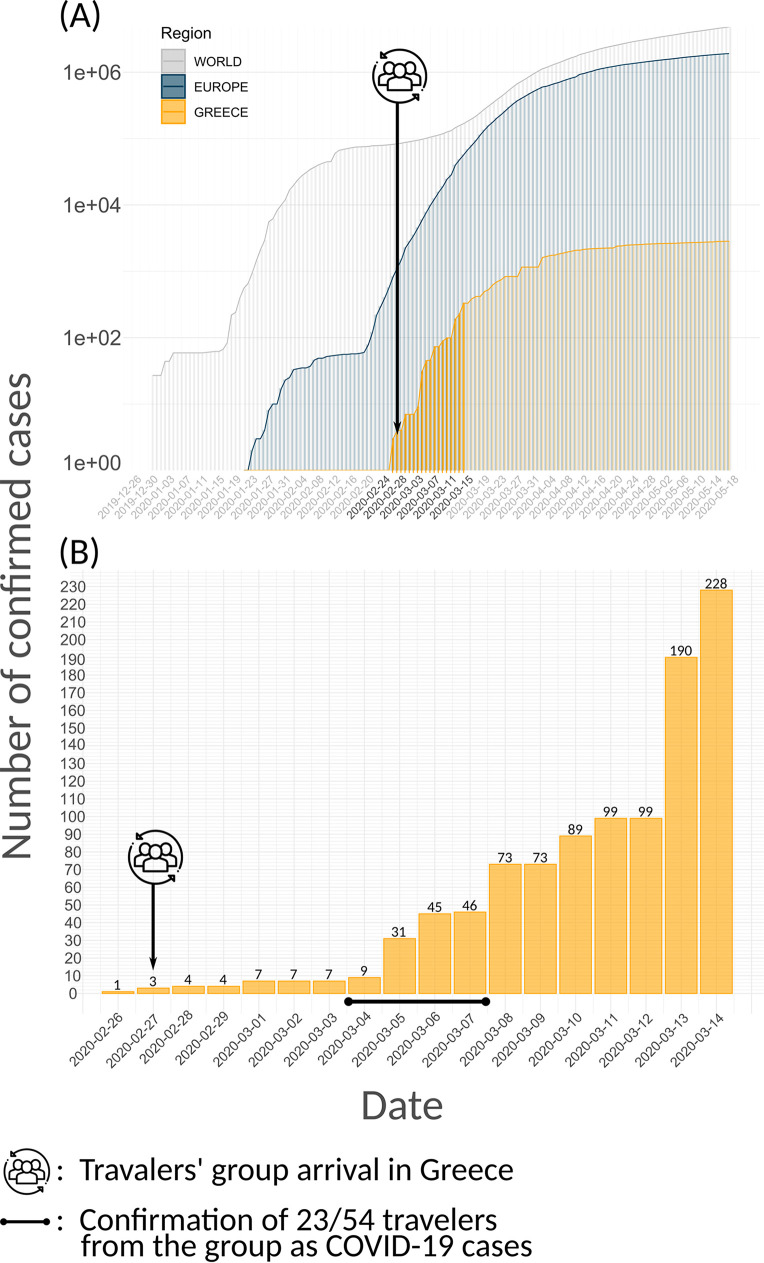
Epidemic curves presenting (A) the log-scaled confirmed cases in the world (gray), Europe (blue), and Greece (yellow) during the first pandemic wave, focusing on the first days of the SARS-CoV-2 pandemic in Greece (Feb 26, 2020 to March 14, 2020) and (B) the confirmed cases in Greece from February 26, 2020 (first confirmed case) to March 14, 2020 (7 days after the travelers’ group arrival in Greece).

As presented in Fig. 1, there was an increase in COVID-19 cases on March 5, 2020 in Greece. Considering that a limited number of tests were performed at the points of entry into the country (6,100 total tests performed up to March 14, 2020 [1]), and thus, new cases would have been imported, molecular epidemiology tools were applied in order to identify the contribution of this first group of travelers to the spread of the virus to the population living in Greece.

### Demographic characteristics of Greece-derived cases and molecular taxonomy of the viral sequences.

**FIG 2:**
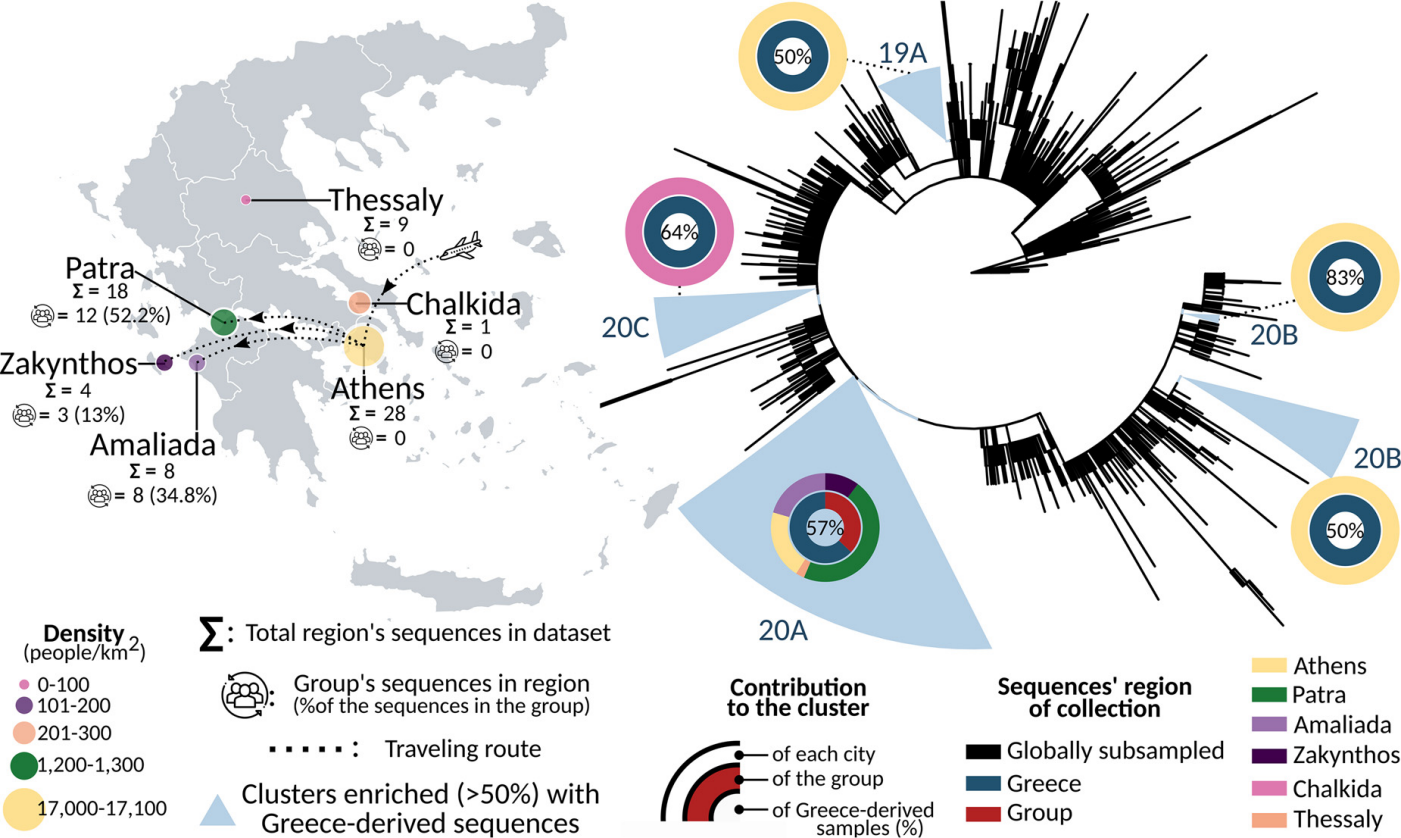
Geographical distribution of the total sequences collected in Greece, along with the proportion of them that were imported from the group that visited Egypt and Israel (left). Maximum likelihood phylogenetic tree consisting of 2,451 unique sequences, 138 (5.63%) of which were collected in Greece, focusing on the clusters (presented in light-blue triangles) that consist of more than five sequences and at least 50% of them are Greece-derived (right). The molecular taxonomy presented for each MTC is based on Nextstrain’s Clade nomenclature. The depicted map was generated in R (Version 4.1.3) (43) after loading the open-source JavaScript library Leaflet version 2.1.1 (47).

Our data set consists of the 23 sequences of SARS-CoV-2 that were isolated from the confirmed cases of the travelers’ group from Egypt and Israel, as well subsampled sequences from multiple regions of Greece, as presented in Table 1 and Fig. 2 and global subsampled sequences. The group’s samples were collected from March 4, 2020 to March 7, 2020 in various hospitals in the hometowns of the infected cases because after their arrival at the Eleftherios Venizelos (Athens) International Airport, they continued their trip back home. Specifically, 12/23 (52.2%) of the group’s cases traveled from Athens to Patra, 8/23 (34.8%) to Amaliada, and 3/23 (13%) to the island of Zakynthos, as presented in Fig. 2. As for the molecular taxonomy of the sequences derived from the group, all of the sequences were identified as B.1 Pango lineage and 20A Nextstrain clade. Regarding the gender of the group’s cases, 17/23 (73.9%) were female and 6/23 (26.1%) were male. A total of 10/23 (43.48%) of the group’s cases fall in the 65 to 100 age group, 8/23 (34.78%) in the 50 to 64 age group, 3/23 (13.04%) in the 35 to 49 age group, and 1/23 (4.35%) in the 20 to 34 age group, while there is also one missing value regarding the age of a female case (Seq.10_GROUP_GR).

### Four distinct genetic variants dominated in Greece during the first days of the pandemic.

As a first step in identifying the role of the imported group in the spread of the virus to the population living in Greece, a phylogenetic tree was generated as described in the Materials and Methods/Alignment, phylogenetic reconstruction, and phylodynamics analysis paragraph. Five clusters consisting of more than five sequences from which at least 50% were collected in Greece were identified. Specifically, 68/138 (49.28%) Greece-derived sequences (presented in Table 1) were found within clusters and 70 sequences were found dispersed in the tree. As presented in Fig. 2, the distinct five clusters were classified under the 19A, 20A, 20B, and 20C Nextstrain clades. The largest cluster consisted of 68 sequences, 39 of which (57.4%) were Greece-derived. All sequences of this cluster are classified as 20A Nextstrain clade and all sequences of the group fall within it. Moreover, the majority of the sequences were collected from the city of Patras (18/39, 46.15%), 8/39 (20.51%) from Athens, 8/39 (20.51%) from Amaliada, 4/39 (10.26%) from the island of Zakynthos, and 1/39 (2.56%) from Chalkida. As for the cluster classified under the 19A clade, 10/20 (50%) sequences were derived from Greece and specifically from the capital city of Athens. The cluster classified under the 20C clade consisted of 14 sequences in total, nine of which (64.29%) are Greece-derived and notably from Thessaly. Finally, two clusters were classified under the 20B clade and were collected from Athens. Of those, the first has 5/10 (50%) and the second 5/6 (83.33%) Greece-derived sequences.

### The imported group affected the course of the pandemic in Greece.

**FIG 3:**
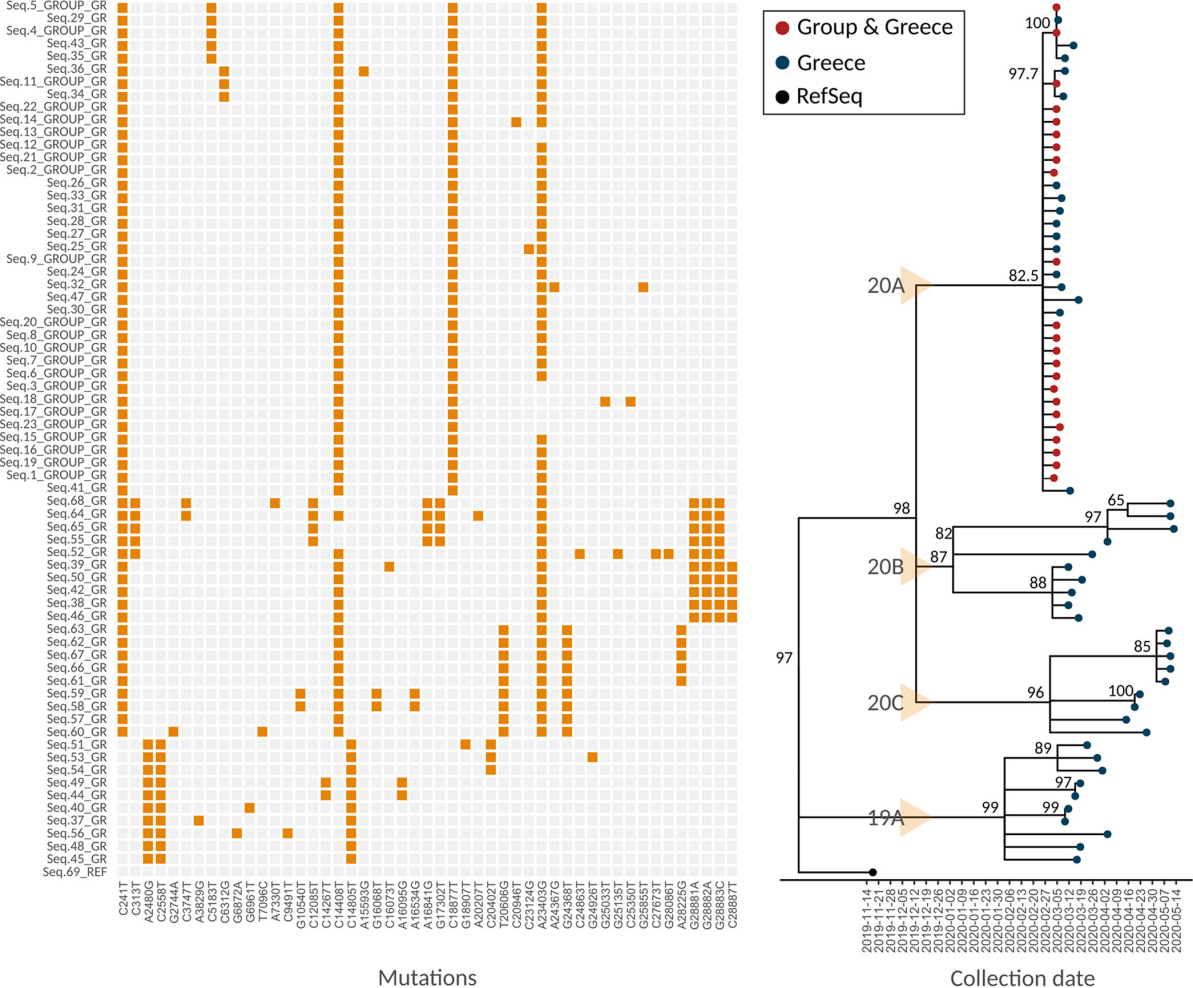
Phylodynamic tree generated with the sequences falling withing Greece-enriched clusters, along with the nucleotide substitution profiles of each sequence depicted as a heatmap. Yellow arrows depict the Nextstrain’s Clade in which the sequences are classified.

The 68 Greece-derived sequences that were found in Greece-enriched clusters, were exported and a second maximum likelihood (ML) phylodynamic tree was generated, as presented in Fig. 3. Four distinct, clearly chronologically defined molecular transmission clusters were identified. The largest molecular transmission cluster (MTC) is classified under the 20A Nextstrain clade and consists of 39 sequences, including all the sequences collected from the imported cases (23/39, 58.97%) and 16 (41.03%) sequences from Greece obtained during the subsampling. Moreover, based on the mutation profiles of the analyzed sequences, four closely related genomic SARS-CoV-2 variants were imported from the group and dispersed into the population living in Greece. The dominant variant (27/39, 69.23%) was the C241T, C14408T, C18877T, A23403G genomic variant that encodes the ORF1b:P314L, S:D614G strain, followed by the C241T, C5183T, C14408T, C18877T, A23403G that encodes the ORF1a:P1640S, ORF1b:P314L, S:D614G strain (5/39, 12.82%), the C241T, C14408T, C18877T that encodes the ORF1b:P314L strain (4/39, 10.26%), and the C241T, C6312G, C14408T, C18877T, A23403G that encodes the ORF1a:T2016R, ORF1b:P314L, S:D614G (3/39, 7.69%). It should be mentioned that the ORF1b:P314L, S:D614G strain is the one that dominated in Greece during the first pandemic wave and molecular data suggest that its first import in Greece was from this group. The strain characterized by the mutation profile C241T, C14408T, C18877T was not dispersed in Greece and it was identified only in the group’s cases. Interestingly, this strain lacks the S:D614G amino acid substitution while the cases from which it was isolated do not harbor a special epidemiological and/or demographic feature (e.g., age group, gender, household location, etc.) that could explain the nondispersal pattern.

### The ORF1b:P314L, S:D614G strain that was first imported from the group and dominated Greece during the first pandemic wave was from Egyptian origin.

**FIG 4:**
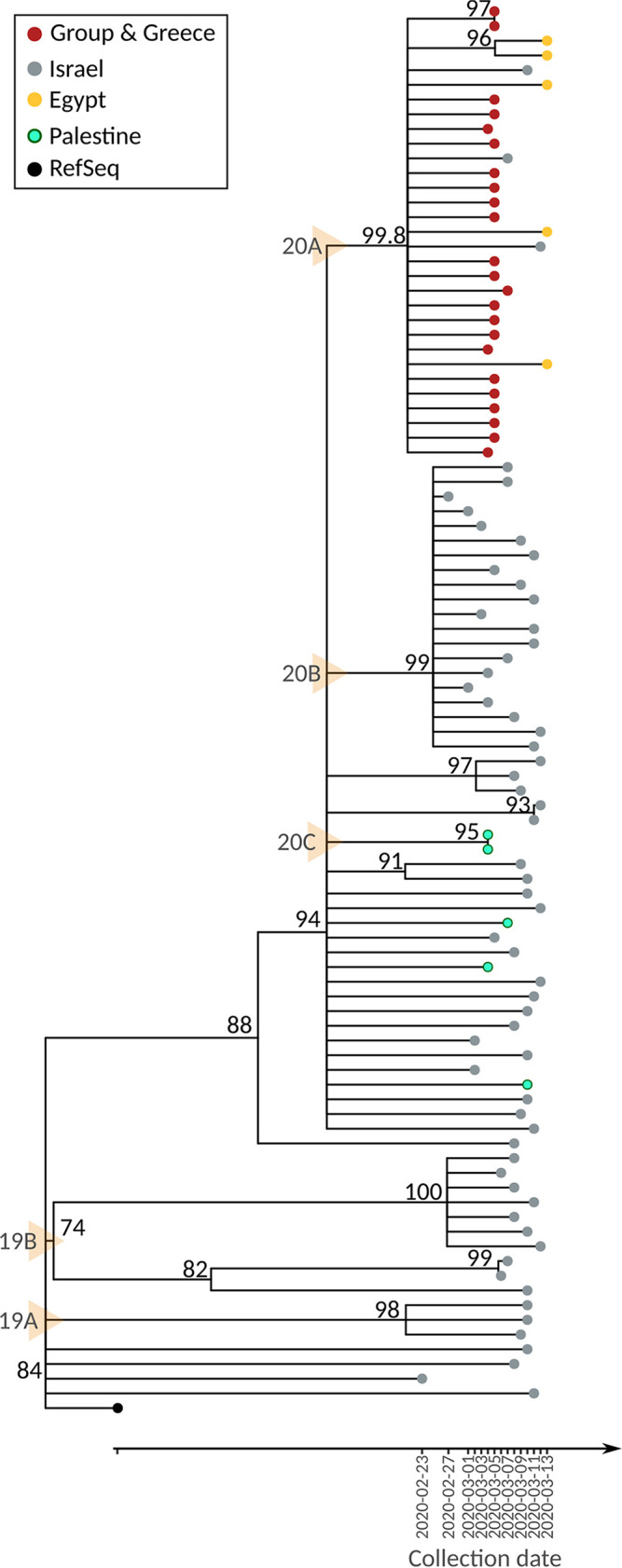
Phylodynamic tree generated from the sequences collected from the cases of the imported group and all of the available sequences from Egypt, Israel, and Palestine from February 23, 2020 to March 13, 2020 (+/– 2 weeks from the collection date of the first group’s sequence). Yellow arrows depict the Nextstrain’s Clade in which the sequences are classified.

Molecular epidemiology and phylodynamics could provide answers regarding the origin of the SARS-CoV-2 strain contracted by the travelers’ group and whether the infection occurred in Egypt—which was the first destination (Mountain Sinai) or in Israel (Holy Land)—the second destination of the group. Thus, we initially investigated the clinical reports of the cases. The onset of symptoms of the first reported case (Seq.1_GROUP_GR) were before his arrival in his hometown (Patras). Considering that SARS-CoV-2 has a mean incubation period of 5.2 days (95% confidence interval [CI] = 4.1 to 7.0) (12), the infection was probably established during the patient’s stay in Egypt. In order to verify this hypothesis, a phylodynamic analysis was performed using the group’s sequences and all of the available sequences in GISAID from Egypt, Israel, and Palestine, collected from February 23, 2020 to March 13, 2020 (±2 weeks of the collection dates of the group’s cases) (Fig. 4). At this point, it should be mentioned that the first reported case in Egypt was on February 14, 2020, in Israel on February 21, 2020, and in Palestine on March 05, 2020. Within the particular time frame in the GISAID database, there were five sequences available from Egypt, 63 from Israel, and five from Palestine. Indeed, all Egypt-derived sequences were found in the same cluster as the group’s sequences along with 3/63 sequences from Israel. However, all of the subsampled sequences that fall in the same subtree as the group’s sequences were collected after the collection date of the group’s sequences. Interestingly, when further investigating the nucleotide substitution profiles of the sequences submitted to the GISAID database within the defined time period (February 23, 2020 to March 13, 2020), we found two sequences with the mutation profile C241T, C14408T, C18877T, A23403G (ORF1b:P314L, S:D614G) that were collected in Taiwan and Lebanon on March 2, 2020 and March 4, 2020, respectively. Both cases, which can be tracked in the GISAID database under the accession IDs EPI_ISL_413592 and EPI_ISL_450508, had travel history to Egypt at the same time period of the group.

## DISCUSSION

The present study focuses on the first days of the SARS-CoV-2 pandemic in Greece when the overall prevalence of SARS-CoV-2 infection was low. We examined whether the SARS-CoV-2 strains initially imported from a group of travelers, who returned to Greece on February 27, 2020, from Egypt (first destination) and Israel (second destination), affected the course of the pandemic in Greece. As testing was not conducted either in the departure (Israel) nor the arrival airport (Athens, Greece), the travelers returned to their hometowns, and 1 week later 23/54 were subsequently identified as confirmed cases. Genomic analysis revealed that four closely related strains were imported from the cases of the group while all of them fall within the B1 lineage. These results are in concordance with previous findings suggesting that in other European countries the dominant lineage at the beginning of the pandemic was lineage B (13–15). Four different strains were identified in a group of people that traveled together, a fact that could be related to different transmission events, intrahost genomic variability, and intrapatient polymorphic quasispecies (16), as well as partially by sequencing bias and homoplasy. Phylogenetics and phylodynamics analyses revealed that three-fourths of these strains spread in the population living in Greece and a molecular transmission cluster of 39 sequences was observed, including the 23 sequences derived from the group’s cases and 16 sequences collected individually. Interestingly, in those three strains that spread, all carried the D614G amino acid substitution in the Spike protein while the strain that didn’t spread does not harbor it. The 614G mutation has been previously associated with increased viral shedding, infectivity, and transmissibility compared with its ancestor 614D (3, 17–19). Of note, in Amaliada, where it is confirmed that the first cases were from the travelers returning to their hometown from Egypt and Israel, 7/8 (87.5%) of the group’s imported strains harbored the 614G mutation and this could partially explain the large increase of COVID-19 cases presented between late February, 2020 and middle March, 2020, testing the resilience of the health care system. Moreover, based on our analysis, the ORF1b:P314L, S:D614G strain that dominated in Greece through the first pandemic wave was initially imported from the group, at the early stage of the pandemic and before the implementation of travel bans. This result complies with previous studies suggesting that successful clusters of transmission during the first pandemic wave were developed by strains that arrived early, several times, and to diverse locations (8). Thus, founder effects early in the pandemic are important for the success of certain variants and the development of large transmission clusters that can be revealed via molecular epidemiological approaches (8, 20). Specifically, during the first pandemic wave in Greece, imported strains were responsible for more than 40% of the transmissions (11), while travel bans had a positive impact in controlling the epidemics as reopening of the borders led to new introductions which gradually converted into local spreads, reflected in the formation of new MTCs (6). These findings, coupled with the high mobility of our connected world, suggest that a variant may end up dominating the epidemic in a particular geographic location, a notion that was also previously proposed for Spain and Boston (8, 21). Taking these into account, we applied a combination of phylogenomics approaches and epidemiological evidence in order to find the origin of the imported strains. Specifically, by tracing the genomic strains circulating the globe in a clearly defined time frame that was chosen on scientific knowledge regarding the incubation time of SARS-CoV-2 (12) and previously known epidemiological and case-report data characterizing the studied group, we could hypothesize that the origin of the ORF1b:P314L, S:D614G imported strain was Egypt. Kostaki et al. (11) revealed that the largest monophyletic cluster of SARS-CoV-2 sequences in Greece dated back to February 15, 2020. As the authors suggest, this could be due to the fact that the virus was circulating in Greece for at least 10 days before it was first detected. However, based on our analysis, this could also be explained by combining the evidence that the dominant strain circulating in Greece during the first wave of the pandemic, that carried the S:D614G mutation, was first imported from the studied group and the first reported case in Egypt was on February 14, 2020. Although neither of these hypotheses can be rejected, given the fact that the dominant strain carried the 614G, it is unlikely to have such an early introduction of a strain with an undetectable, low-level transmission. Conclusively, SARS-CoV-2 is the first pandemic in history monitored almost in real-time through genomic analysis of strains around the world (22). Research conducted worldwide generated valuable data regarding the role of imported cases, in a world described by high mobility, the need for prioritization of the NPIs, immediate response with health screening procedures, especially at the airports and alternative entry points, and the development of robust surveillance systems that trace the viral transmission dynamics and provide representative data sets from multiple regions of each country. We believe that our results enlighten the events that took place during the first days of the SARS-CoV-2 pandemic in Greece with the import of the studied group, while highlighting the limitations arising from the under-representation of genomics data from specific countries and the heterogeneous sampling across different regions of the same country.

## MATERIALS AND METHODS

### Sampling, sequencing, and consensus calling.

Extraction of the viral genomic RNA was performed using the automated platform NucliSens EasyMag (bioMérieux, Marcy l’Etoile, France) according to the manufacturer’s instructions. Quantification of total RNA was performed with the Qubit fluorimeter and the compatible Qubit RNA HS Assay (Thermo Fisher Scientific, Waltham, MA, USA). Amplicon-based NGS libraries were constructed using the QIAseq SARS-CoV-2 Primer Panel (Qiagen) quality-controlled using the Agilent High Sensitivity DNA assay for Bioanalyzer 2100 (Agilent Technologies), equimolarly mixed, and sequenced in a NextSeq 500 System (Illumina). The generated FASTQ files were aligned to the reference SARS-CoV-2 genome from Wuhan, China (GenBank Accession: NC_045512.2, GISAID Accession: EPI_ISL_402123) with the Bowtie2 aligner (23) using the very-sensitive preset. Consensus FASTA sequences were exported from the generated BAM files using bcftools (24). Sequences were further filtered and those that were incomplete (sequence length < 29,000 nt) or had low coverage (entries with >5% Ns) were filtered out of the analysis.

### Subsampling.

All of the available sequence data and corresponding metadata that had been collected up to May 14, 2020 were downloaded from the GISAID database (25) on January 28, 2022. Sequences that were incomplete (sequence length < 29,000 nt), had low coverage (entries with >5% Ns), or their complete collection dates were absent were filtered out.

### Alignment, phylogenetic reconstruction, and phylodynamics analysis.

Sequences were aligned against the SARS-CoV-2 reference using the MAFFT aligner (26). Specific regions that have been reported to be problematic (27, 28) were masked using the “augur mask” command that is available from Nextstrain’s “augur” pipeline (29). The alignment was manually curated using the aliview software (30) and duplicated sequences were removed, resulting in 2,451 unique sequences of which 138 (5.63%) sequences were collected in Greece. The ML phylogeny reconstruction, the bootstrap analysis, and the SH test were implemented by IQ-TREE (31, 32) by applying 1,000 ultra-bootstrap values. ML phylogenies were estimated using the GTR+F+I substitution model, as suggested by IQ-TREE (33). Finally, the ML phylodynamics analysis was performed with IQ-TREE LSD2 software (34). The nucleotide substitution rate of 8 × 10^−4^ substitutions per site per year was used as previously defined (35, 36). In addition to the SARS-CoV-2 reference genome, we tested 10 more basal sequences that could act as an “anchor” for the phylogeny (GISAID accessions: EPI_ISL_402130, EPI_ISL_402128, EPI_ISL_402121, EPI_ISL_416329, EPI_ISL_451326, EPI_ISL_416907, EPI_ISL_406798, EPI_ISL_457733, EPI_ISL_430742, EPI_ISL_430729) (8). No substantial differences were observed in the tree topologies with the alternative roots; thus, all phylogenies presented are rooted in the SARS-CoV-2 reference (GenBank accession: NC_045512.2, GISAID accession: EPI_ISL_402123).

### Molecular transmission clusters identification.

Molecular transmission clusters (MTCs) of sequences derived from Greece were identified by combining the approaches described by Paraskevis et al. (37) and López et al. (8). Initially, a phylogenetic tree consisting of 2,451 unique sequences, 138 (5.63%) of which were collected in Greece, was generated as described previously. We searched for clusters that consisted of more than five sequences where at least 50% of them were collected in Greece. Greece-derived sequences that fell within these clusters were exported and a second ML phylodynamic tree was generated, rooted to the SARS-CoV-2 reference. MTCs identification was performed using two criteria: clusters with maximum genetic distance ≤ 0.005 and bootstrap support value > 80 (phylogenetic confidence criterion).

### Mutation profiles and lineage/clade assignment.

NextClade CLI (Version 1.11.0) (38) was used in order to perform quality control (QC) of the FASTA sequences, assignment to a Nextstrain clade and nucleotide substitution profile, and identification of changes in the viral proteins relative to the SARS-CoV-2 reference. Nextstrain’s molecular taxonomy nomenclature is based on a year-letter clade assignment when (i) a clade reaches >20% global frequency for 2 or more months; (ii) a clade reaches >30% regional frequency for 2 or more months; or (iii) a variant of concern (VOC) (39) is recognized (40). In order to examine the phylogenetic structure of SARS-CoV-2 at higher resolution, the Pango nomenclature (15) was also used for classification purposes. More than 1,300 Pango lineages (https://cov-lineages.org [41]) have been described, covering the entire genetic diversity of SARS-CoV-2, some of which are genetically very similar to each other and are intended to represent “epidemiologically relevant” events. Pango lineage assignment was performed via the Pangolin Toolkit (Version 3.1.11) (13).

### Epidemiological data.

Epidemiological data, including cases and deaths per day per country and daily number of tests performed, were downloaded (March 18, 2022) from the ECDC (1) and loaded into R via the tidycovid19 package (Version 0.0.0.9000) (42).

### Statistical analysis.

All statistical analyses were performed in R (Version 4.1.3) (43). Packages ape (Version 5.6.2) (44), tidyverse (Version 1.3.1) (45), tidycovid19 (Version 0.0.0.9000) (42), and ggplot2 (Version 3.3.6) (46) were used for the phylogenetic and phylodynamic trees and metadata manipulation and visualization.

### Ethics statement.

All procedures performed during the present study were in accordance with the 1964 Helsinki Declaration and its later amendments or comparable ethical standards.

### Data availability.

The findings of this study are based on molecular data and corresponding metadata associated with sequences available on GISAID, at gisaid.org, submitted up to January 28, 2022. The sequences originating from Greece are available on GISAID, at gisaid.org, under the Accession IDs presented in Table 1.
